# Diabetes Downregulates Allergen-Induced Airway Inflammation in Mice

**DOI:** 10.1155/2018/6150843

**Published:** 2018-04-15

**Authors:** Vinicius F. Carvalho, Emiliano O. Barreto, Ana Carolina S. Arantes, Magda F. Serra, Tatiana Paula T. Ferreira, Yago A. P. Jannini-Sá, Cory M. Hogaboam, Marco A. Martins, Patrícia M. R. Silva

**Affiliations:** ^1^Laboratory of Inflammation, Oswaldo Cruz Institute, FIOCRUZ, Av. Brasil No. 4365, 21045-900 Manguinhos, RJ, Brazil; ^2^Federal University of Alagoas, 57072-970 Maceió, AL, Brazil; ^3^Cedars-Sinai Medical Center, 8700 Beverly Blvd, Los Angeles, CA 90048, USA

## Abstract

Previous studies described that allergic diseases, including asthma, occur less often than expected in patients with type 1 diabetes. Here, we investigated the influence of diabetes on allergic airway inflammation in a model of experimental asthma in mice. Diabetes was induced by intravenous injection of alloxan into 12 h-fasted A/J mice, followed by subcutaneous sensitization with ovalbumin (OVA) and aluminum hydroxide (Al(OH)_3_), on days 5 and 19 after diabetes induction. Animals were intranasally challenged with OVA (25 *μ*g), from day 24 to day 26. Alloxan-induced diabetes significantly attenuated airway inflammation as attested by the lower number of total leukocytes in the bronchoalveolar lavage fluid, mainly neutrophils and eosinophils. Suppression of eosinophil infiltration in the peribronchiolar space and generation of eosinophilotactic mediators, such as CCL-11/eotaxin, CCL-3/MIP-1*α*, and IL-5, were noted in the lungs of diabetic sensitized mice. In parallel, reduction of airway hyperreactivity (AHR) to methacholine, mucus production, and serum IgE levels was also noted under diabetic conditions. Our findings show that alloxan diabetes caused attenuation of lung allergic inflammatory response in A/J mice, by a mechanism possibly associated with downregulation of IgE antibody production.

## 1. Introduction

Asthma is a chronic inflammatory disease of the airways that reduces lung function and induces airway hyperresponsiveness (AHR) to nonspecific irritants [1, 2]. AHR is defined as an exacerbation of the airway responses towards stimulation, by a mechanism associated with a direct effect of inflammatory mediators on the airway smooth muscle and/or indirectly dependent on neural pathways or mast cell activation [3]. Different from allergic inflammatory responses, AHR does not depend exclusively on antigen activation and can result from nonspecific stimulation with irritants, including cold air, fumes or smoke, and particles [4, 5]. Currently, there is a controversy in the association of asthma and type 1 diabetes. While several studies have shown a reduced incidence of asthma in type 1 diabetic patients [6, 7], others demonstrated an increased intercurrence between these diseases [8, 9]. In the works reporting a lower incidence of asthma in type 1 diabetic patients, the physiopathological mechanisms associated with this epidemiological profile are not yet fully understood [6, 7].

The immune and inflammatory responses in allergic asthma are associated with specific immunoglobulin E (IgE) and involve many target cells, including mast cells, CD4^+^ T lymphocytes, and eosinophils [2]. The establishment of IgE-bearing cells, such as mast cells and basophils, sets the stage for their activation in a subsequent exposure to allergen. The mediators released are responsible for vascular permeability increase and recruitment and also for AHR [10, 11]. Eosinophils release cytotoxic mediators as basic proteins, including eosinophil cationic protein (ECP) and eosinophil peroxidase (EPO), and reactive oxygen species in the airways causing mucosal damage. Also, they produce cytokines and lipid mediators exacerbating the inflammatory response inducing airway remodeling [12]. IL-5, which is a central T_H_2 cytokine with a key role in eosinophil growth, maturation, differentiation, survival, and activation [13], shows elevated levels in the bronchoalveolar fluid and serum of asthmatic patients [14, 15]. Of note, chemokines such as CCL-3/MIP-1*α*, CCL-5/RANTES, CCL-7/MCP-3, CCL-8/MCP-2, CCL-11/eotaxin-1, CCL-13/MCP-4, and CCL-24/eotaxin-2 have been found to induce the eosinophil migration and activation in inflammatory diseases [12].

We and others have shown that alloxan-diabetic rats when sensitized and challenged with antigen show lower levels of eosinophil infiltration in the pleural space [16] and bronchoalveolar lavage fluid [17], a response which paralleled with reduced local mast cell population [18, 19]. Under the condition of mast cell adoptive transfer to diabetic rats, the pleural allergic inflammatory response was restored, indicating that the downregulation of mast cells could be responsible for the lack of responsiveness of diabetic animals to antigen challenge [20]. Taking the previous observations into consideration, this study was undertaken to investigate the influence of the diabetic state on the tissue eosinophil recruitment and AHR in a murine model of allergic asthma.

## 2. Methodology

### 2.1. Animals

Male A/J mice (18–20 g) were obtained from the Oswaldo Cruz Foundation breeding. All procedures involving care and use of laboratory animals in this study were examined and approved by the Animal Ethics Committee of the Oswaldo Cruz Foundation (license LW 23/11). Mice were housed in groups of six in a temperature-, humidity-, and light-controlled (12 h light : 12 h darkness cycle) colony room. Mice were given ad libitum access to food and water. Twelve animals were randomly assigned into 2 groups as follows: nondiabetic (*n* = 6) and diabetic (*n* = 6), for quantification of insulin and corticosterone levels. In another set of experiments, 24 mice were randomly assigned into 4 groups as follows: nondiabetic control (*n* = 6), nondiabetic allergen-challenged (*n* = 6), diabetic control (*n* = 6), and diabetic allergen-challenged (*n* = 6). All analyses were repeated twice.

### 2.2. Diabetes Induction

**Figure 1:**
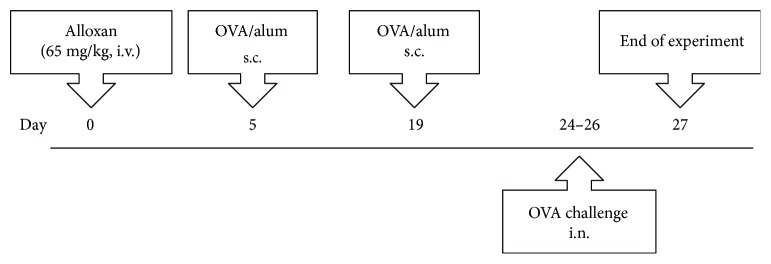
Brief scheme of animal sensitization and challenge with ovalbumin (OVA). i.v.: intravenous; s.c.: subcutaneous; i.n.: intranasal.

Diabetes mellitus was induced by an intravenous (i.v.) injection of 65 mg/kg of alloxan monohydrate dissolved in sterile saline (0.9%, NaCl). Control mice were injected with sterile saline only. The presence of diabetes was verified by blood glucose concentration > 11.2 mmol/L, determined with a blood glucose monitor in samples obtained from a cut tip of the tail. Blood glucose levels were measured immediately before the experiments. All the analyses were performed 27 days after alloxan injection (Figure 1).

### 2.3. Active Sensitization and Challenge

After diabetes induction, mice were sensitized on days 5 and 19 with a subcutaneous injection of 50 *μ*g ovalbumin (OVA), emulsified in 5 mg aluminum hydroxide (Al(OH)_3_) to total volume of 0.2 mL, followed by intranasal challenge with OVA droplet aspiration (25 *μ*g/25 *μ*L of saline), once a day for 3 consecutive days (days 24–26) (Figure 1). Control mice received instillation with sterile saline. In the case of ovalbumin-specific immunoglobulin analyses, the control mice were sham-sensitized with sterile saline and received challenge with ovalbumin. Analyses were performed 24 h after the last instillation.

### 2.4. AHR Evaluation

Animals were placed conscious and unrestrained in cylindrical plexiglass single chambers that were connected to a barometric whole-body plethysmography (Buxco Research System, Wilmington, NC, USA) for measuring the enhanced pause (Penh) responses in conscious, spontaneously breathing mice following appropriate provocations. Mice were aerosolized with phosphate-buffered saline (PBS) and methacholine (3 and 6 mg/mL) through an inlet of the individual chambers for 2.5 min, and Penh readings were recorded for 5 min following each nebulization. AHR was expressed as Penh, an indirect measurement that is correlated with airway resistance, impedance, and intrapleural pressure.

### 2.5. Bronchoalveolar Lavage (BAL)

Mice were euthanized by terminal anesthetic overdose (sodium thiopental, 500 mg/kg, i.p.), and the bronchoalveolar lavage (BAL) was performed with 2 × 0.75 mL of PBS plus ethylenediaminetetraacetic acid (EDTA) (10 mM) through the tracheal cannula. The BAL was centrifuged (170 *×*g for 10 min at 20°C), the pellet resuspended in 1 mL of PBS, and cells (90 *μ*L) were stained with 0.2% crystal violet (10 *μ*L). Total cell number was determined by light microscopy (BX40; Olympus) in a Neubauer chamber. Differential cell counts were carried out in cytocentrifuged preparations stained with May-Grünwald Giemsa dye, under oil immersion objective to determine the percentage of mononuclear cells, eosinophils, and neutrophils.

### 2.6. Histological Studies

Twenty-four hours after the last challenge, the lungs were perfused with Millonig buffer solution (pH = 7.4), with 4% paraformaldehyde, and removed. Routine histological procedures were used to paraffin-embedded samples, and 4 *μ*m sections were stained with hematoxylin and eosin (H&E). Mucus-secreting goblet cells were visualized by staining with periodic acid-Schiff (PAS). The analysis was performed under a light microscope (BX50; Olympus) coupled to a video camera (Optronics Engineering, DEI-750). Images were ultimately analyzed using image analyzer software (Image-Pro Plus 4). The camera output was processed and analyzed by the image analyzer software Image-Pro Plus 4. PAS staining was quantified by densitometric scanning (pixels)/*μ*m^2^.

### 2.7. Evaluation of Lung Eosinophils

After performing BAL, the lungs were perfused with calcium- and magnesium-free Hank's balanced salt solution (HBSS) containing 10% fetal calf serum (FCS), 0.6 mM EDTA, 100 U/mL penicillin, and 100 mg/mL streptomycin via the right ventricle at a rate of 4 mL/min for 5 min. Lungs were removed, cut, and incubated in 4 mL of HBSS containing 175 U/mL collagenase, 10% FCS, 100 U/mL penicillin, and 100 mg/mL streptomycin and incubated for 60 min at room temperature. After digestion, the free cells were resuspended in HBSS and counted in Neubauer chamber, and cytospin slides were made. Slides were stained with May-Grünwald Giemsa and eosinophils counting using a light microscope (BX50; Olympus).

### 2.8. Measurement of Cytokine Levels in Lung Tissue

ELISA was used to measure IL-5, CCL-3/MIP-1*α*, and CCL-11/eotaxin-1 in lung homogenates tissue. Lungs were homogenized in PBS (1 mL/50 mg of lung tissue), in ice, and centrifuged at 12.000 ×g for 15 min, and the supernatant was stored at −80°C until use. The samples were assayed using commercial ELISA kits according to manufacturer's instructions (R&D Systems).

### 2.9. Ovalbumin-Specific Immunoglobulin E Quantification

OVA-specific serum IgE levels were determined by ELISA using serum obtained from blood samples collected 24 h after the last OVA challenge. In brief, a 96-well plate was coated with OVA (10 mg/mL) and then treated with mouse serum followed by biotin-conjugated rat anti-mouse IgE. In addition, duplicate twofold dilutions of an OVA-specific IgE reference serum (made from subcutaneous OVA-sensitized mice) were also added, with a 1 : 10 starting dilution, in order to create a standard curve. Then, avidin-horseradish peroxidase (HRP) solution was added to each well. Units are optical density readings at 405 nm.

### 2.10. Hormone Evaluation

Twenty-seven days after diabetes induction, animals were euthanized in a CO_2_ chamber, during the nadir (08:00 h) of the circadian rhythm as described previously [21], and blood was immediately collected from abdominal aorta in vials containing heparin (40 U/mL) for insulin and corticosterone quantification. Plasma was obtained after sample centrifugation at 1000 *×*g, for 10 min and stored at −80°C until use. Insulin and corticosterone levels were quantified by radioimmunoassay following manufacturer's instructions (MP Biomedicals, Solon, OH, USA), in a gamma counter (ICN Isomedic 4/600 HE; ICN Biomedicals Inc., Costa Mesa, CA, USA).

### 2.11. Drugs

Alloxan monohydrate, ovalbumin, methacholine, streptomycin, penicillin, EDTA, paraformaldehyde, and HBSS were purchased from Sigma Chemical Co (St. Louis, MO, USA); collagenase A and fetal calf serum (FCS) from Invitrogen (CA, USA). All solutions were freshly prepared immediately before use.

### 2.12. Data Analysis

All data were presented as mean ± SEM. For comparison of two groups, we statistically analyzed by unpaired *t*-test, while the comparison of three or more groups was analyzed by one-way ANOVA, followed by the Newman-Keuls Student's *t*-test multiple comparisons test. Probability values (*P*) of 0.05 or less were considered significant.

## 3. Results

### 3.1. Characteristics of Alloxan-Induced Type 1 Diabetes in A/J Mice

Alloxan-diabetic mice presented increased blood glucose levels with a simultaneous decrease in body weight and plasma insulin levels, clearly indicating a type 1 diabetic state. There was also an increase in the plasma corticosterone levels in diabetic as compared to nondiabetic mice (Table 1).

### 3.2. Diabetes Inhibits Allergen-Induced Airway Inflammation

**Figure 2:**
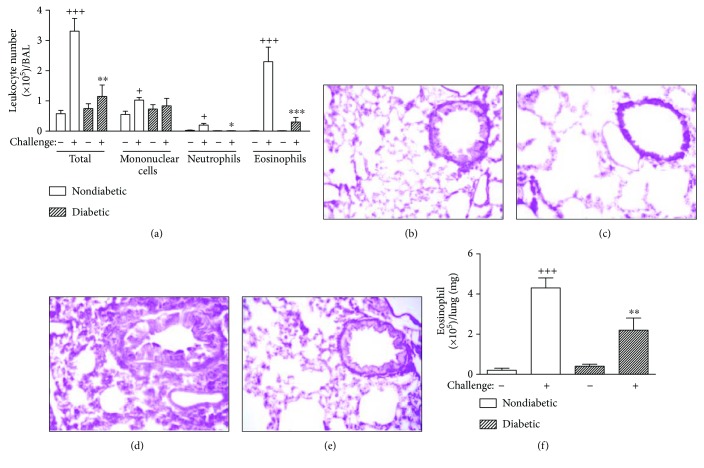
Alloxan diabetes attenuates OVA-induced airway inflammation in lung tissue of sensitized mice. Profile of cells in BAL and lung tissue in diabetic allergen-challenged mice. (a) Total cell counts were determined on 1.5 mL BAL, and differential cell counts were assessed by May-Grünwald Giemsa staining. (b–e) Lung sections were stained with hematoxylin and eosin (H&E) for measurement of inflammatory cells around the airways. Data revealed a different extent of cellular infiltration of the peribronchiolar area. Sections were obtained from lungs of nondiabetic saline-challenged mice (b), diabetic saline-challenged mice (c), nondiabetic OVA-challenged mice (d), and diabetic OVA-challenged mice (e). Lungs were removed 24 h after the last challenge. Six animals were assigned to each group. Scale bar 100 *μ*m. (f) Eosinophil count in lung tissue from nondiabetic or diabetic mice challenged with saline or ovalbumin (OVA). The analysis proceeded 24 h after the last OVA challenge. Data were expressed as mean ± SEM (*n* = 6). ^+^*P* < 0.05 and ^+++^*P* < 0.001 versus nondiabetic saline-challenged mice; ^∗^*P* < 0.05, ^∗∗^*P* < 0.01 and ^∗∗∗^*P* < 0.001 versus nondiabetic OVA-challenged mice.

We examined the effects of alloxan-induced diabetes on a model of allergic lung inflammation triggered by OVA in sensitized mice. Twenty-four hours after the last challenge, the cellular component in BAL was analyzed. In nondiabetic sensitized and saline-challenged mice, no increase in the leukocyte content of BAL was evidenced. As expected, after sensitization and challenge with OVA, the numbers of mononuclear cells, neutrophils, and eosinophils in BAL were significantly increased in nondiabetic animals. Nevertheless, under conditions of alloxan diabetes, a reduction in the eosinophil and neutrophil numbers in BAL was noted after OVA, although mononuclear cells were not affected (Figure 2(a)).

Histological examination of H&E-stained lung sections from nondiabetic sensitized and OVA-challenged mice showed a large number of infiltrating leukocytes in the peribronchiolar space (Figure 2(d)), in comparison with saline-challenged mice (Figure 2(b)). Diabetic sensitized OVA-challenged mice showed a marked decrease in the leukocyte infiltration (Figure 2(e)) compared to nondiabetics (Figure 2(d)). Additionally, allergen-induced eosinophil accumulation was evaluated in lung tissue sections after digestion with collagenase by staining with May-Grünwald Giemsa. As illustrated in Figure 2(f), diabetic sensitized OVA-challenged mice showed a less pronounced eosinophil accumulation when compared to nondiabetic sensitized challenged mice.

**Figure 3:**
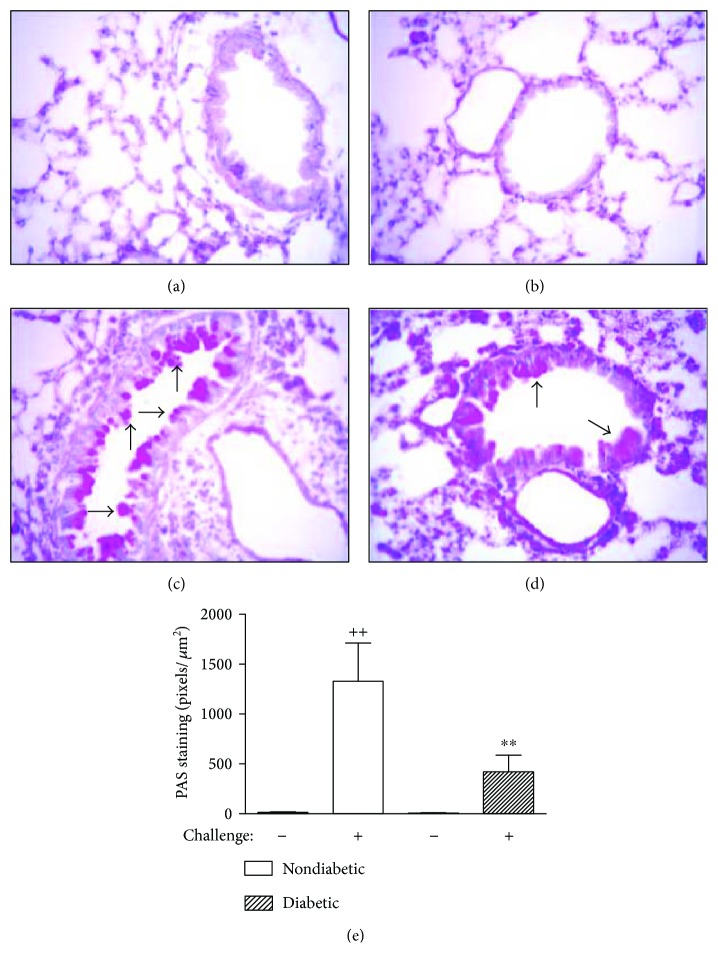
Alloxan diabetes attenuates lung PAS staining of goblet cells in the airways of OVA-induced lung inflammation in mice. Panels (a–d) show photomicrographs of PAS staining (arrows) of lung sections from nondiabetic saline-challenged, diabetic saline-challenged, nondiabetic OVA-challenged, and diabetic OVA-challenged mice, respectively. In (e), quantification of PAS staining in lungs from nondiabetic and diabetic mice. Each value indicates the mean ± SEM from six animals per group. ^++^*P* < 0.01 versus nondiabetic saline-challenged mice; ^∗∗^*P* < 0.01 versus nondiabetic OVA-challenged mice. Scale bar 100 *μ*m.

Lung tissue sections were examined for mucus by means of PAS staining. Nondiabetic sensitized and OVA-challenged mice revealed a strong labeling of cells inside the airways as compared to nondiabetic sensitized saline-challenged mice. Diabetic sensitized mice exhibited a clearly less mucus formation as compared with nondiabetic sensitized when challenged with OVA (Figure 3). Quantitative data are seen in Figure 3(e).

### 3.3. Diabetes Decreases Lung Cytokine and Chemokine Levels in a Murine Model of Asthma

**Figure 4:**
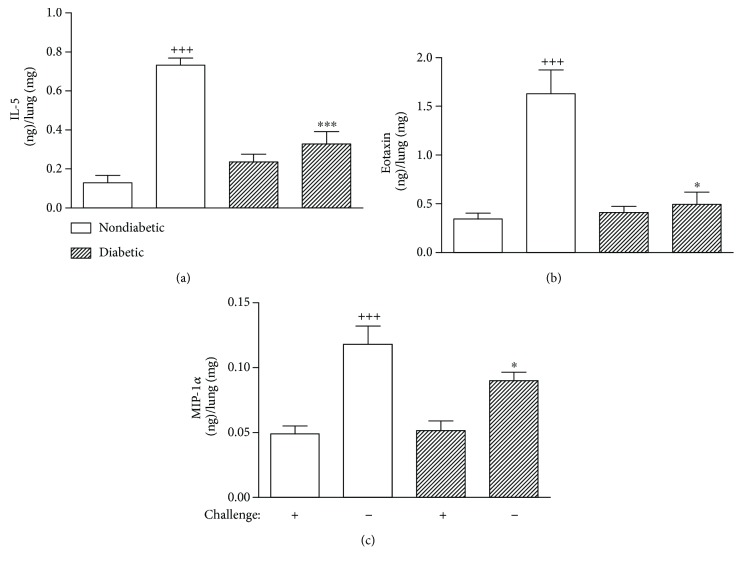
Alloxan diabetes suppressed cytokine production in the lung tissue of sensitized OVA-challenged mice. IL-5 (a), CCL-11/eotaxin (b), and CCL-3/MIP-1*α* (c) production in lung tissue from nondiabetic or diabetic mice challenged with saline or ovalbumin (OVA) was analyzed by ELISA. The analysis proceeded 24 h after the last OVA challenge. Each value indicates the mean ± SEM from six animals per group. ^+++^*P* < 0.001 versus nondiabetic saline-challenged mice; ^∗^*P* < 0.05 and ^∗∗∗^*P* < 0.001 versus nondiabetic OVA-challenged mice.

Nondiabetic sensitized mice when challenged with OVA showed an increase in the levels of IL-5, CCL-3/MIP-1*α*, and CCL-11/eotaxin-1 in lung tissue when compared to those challenged with saline (Figure 4). A significant reduction in the levels of IL-5, CCL-3/MIP-1*α*, and CCL-11/eotaxin was observed in the lungs of allergen-challenged mice under condition of diabetes. No difference in the basal levels of these cytokines was noted in diabetic sensitized animals challenged with saline as compared to the nondiabetics (Figure 4).

### 3.4. Diabetes Reduces Allergen-Specific Immunoglobulin E Levels in the Serum

**Figure 5:**
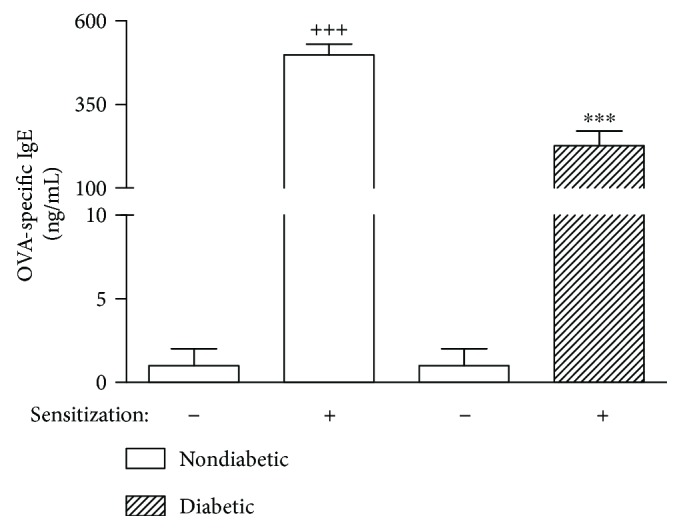
Alloxan diabetes reduces serum OVA-specific IgE antibody levels in mice sensitized with ovalbumin. The levels of OVA-specific IgE were measured at 24 h after the last allergen challenge. Blood was collected by cardiac puncture for measurement of OVA-specific IgE by ELISA. Each value indicates the mean ± SEM from six animals per group. ^+++^*P* < 0.001 versus nondiabetic nonsensitized group. ^∗∗∗^*P* < 0.001 versus nondiabetic sensitized group.

According to ELISA measurements, OVA-specific serum IgE levels in nondiabetic mice sensitized and challenged with OVA were significantly increased as compared to sham-sensitized challenged with OVA. This increase was significantly smaller in diabetic mice sensitized and challenged with OVA (Figure 5).

### 3.5. Diabetes Suppresses the Development of AHR

**Figure 6:**
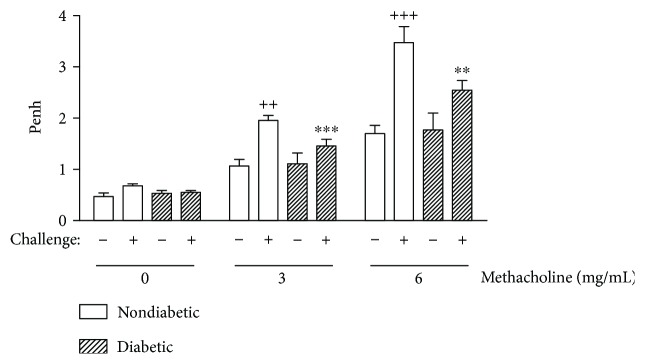
Alloxan diabetes attenuates AHR caused by OVA challenge in sensitized mice. The AHR to aerosolized methacholine was measured 24 h after the last allergen challenge in unrestrained conscious mice. Mice were placed in the main chamber and aerosolized first with PBS followed by increasing doses of methacholine (3 and 6 mg/mL), 2 min for each aerosolization. Readings of breathing parameters were taken for 5 min after each nebulization during which Penh values were determined. Each value indicates the mean ± SEM from five animals per group. ^++^*P* < 0.01 and ^+++^*P* < 0.001 versus nondiabetic saline-challenged mice; ^∗∗^*P* < 0.01 and ^∗∗∗^*P* < 0.001 versus nondiabetic OVA-challenged mice.

Further, we investigated whether alloxan diabetes could affect the development of AHR triggered by OVA in sensitized mice. Twenty-four hours after the last challenge, AHR was assessed by noninvasive whole-body plethysmography. Nondiabetic mice sensitized and challenged with OVA showed an increased Penh to 3 and 6 mg/mL of methacholine aerosolization as compared to sensitized mice challenged with saline. In the case of diabetes, sensitized OVA-challenged mice exhibited a marked attenuation of the increased values of Penh, for both concentration of methacholine, compared to nondiabetic sensitized OVA-challenged animals (Figure 6). No alteration of Penh was detected after PBS aerosolization.

## 4. Discussion

In this study, we investigated the influence of the alloxan-induced diabetic state on the development of experimental allergic asthma, which is induced after allergen sensitization and intranasal challenge with ovalbumin. We found that the eosinophil accumulation in the BAL and in the lungs after allergen challenge was significantly diminished in diabetic mice, with a strong correlation with the decreased levels of IL-5, CCL-11/eotaxin, and CCL-3/MIP-1*α* in lung tissue. OVA-specific serum IgE levels were abrogated in diabetic sensitized animals in comparison with nondiabetic sensitized animals. In addition, diabetic mice presented reduced AHR to methacholine after allergen challenge when compared to nondiabetic mice. Our findings indicate that alloxan-diabetic mice were less susceptible to developing allergen-induced inflammatory response of the airways.

Furthermore, we showed that injection of alloxan increased blood glucose in parallel to a decrease in the body weight and plasma insulin levels, indicating a clear type 1 diabetic state in A/J mice. Also, alloxan-induced type 1 diabetes also led to an increase in circulating levels of corticosterone as compared to nondiabetic animals. These data are in agreement with our previous findings showing that diabetic animals present a hyperactivity of hypothalamus-pituitary-adrenal (HPA) axis with consequent hypercorticoidism [21].

The association between type 1 diabetes mellitus and asthma is still a point of controversy. There are some reports showing a higher incidence of asthma in type 1 diabetic patients [5, 6]; however, others show that allergic diseases, including asthma, occur less often than expected in type 1 diabetic patients [6, 7]. This might reflect differences in geographic location, which can lead to different allergen exposure and dietetic habits, as well as clinical characteristics of the patients evaluated including a good metabolic control. In a previous study, we demonstrated that diabetic rats were refractory to allergen challenge in different systems including an allergen-induced inflammatory response in the pleural cavity and skin [22, 23]. Herein, we showed that sensitized mice exhibited accumulation of inflammatory cells, with the predominance of eosinophils, in both BAL and lung tissue, 24 h after the last intranasal administration of OVA. In the case of diabetic sensitized allergen-challenged mice, BAL and lung eosinophil infiltration were significantly less intense. This response may not be attributable to an intrinsic abnormality in the locomotory function of these cells, since we previously demonstrated that eosinophils recovered from diabetic animals showed no alteration in the locomotory function evaluated *in vitro* [24]. Accumulation of eosinophils in asthmatic patients was shown to be associated with an increase in cell recruitment and reduction of apoptosis [25, 26]. As we noted that plasma corticosterone levels were increased under the condition of alloxan-induced diabetes, this could be accounted for by the reduction of eosinophil infiltration observed in diabetic allergen-challenged mice, in a mechanism associated with glucocorticoid-induced apoptosis and with downregulation of *β*2-integrin-induced leukocyte adhesion [27]. Remarkably, diabetic mice also presented a reduction in the neutrophil influx in the BAL. In contrast to what was noted in the case of eosinophils, glucocorticoids were reported to increase survival of neutrophils [28]. Thus, elevation of corticosterone levels in diabetic mice does not seem to account for the decrease in allergen-induced neutrophil infiltration in the BAL noted in diabetic mice. One possible explanation is the hyperglucagonemia that is present in diabetic animals [29]. In line with this idea, we demonstrated that glucagon inhibited LPS-induced neutrophil influx and TNF-*α* production in BAL of mice [30].

Different from what was observed in case of the granulocytes, no difference was detected in the mononuclear cells in the BAL of diabetic sensitized challenged mice as compared to the nondiabetics. Glucocorticoid hormones do not alter differentiation and responsiveness of Th17 cells [31], though the major subtype of effector lymphocyte involved in the pathogenesis of severe asthma is the CD4 T cells [32]. Because mononuclear cell counts embrace both macrophages and lymphocytes, further studies are now underway to clarify the effect of diabetic state on these leukocyte subtypes. It is noteworthy that diabetic animals show marked thymocyte depletion [33] in association with a reduction in the number of lymphocytes in the peripheral blood [34]. This lymphopenia observed in diabetics may be due to the occurrence of high levels of apoptosis [35] and less proliferation of lymphocytes [36].

Furthermore, we found that the mucus occlusion of the airway lumen was lower in diabetic mice after antigen stimulation as compared to nondiabetic mice. In accordance with our results, lower mucin content was observed in the intestine of diabetic animals [37]. It is noteworthy that histamine released by mast cell degranulation is associated with increased luminal mucus in experimental asthma [38]. In addition, we previously demonstrated that diabetic animals showed a reduction in the mast cell numbers and reactivity [19, 22], which could be considered as a plausible explanation for the reduction in the allergen-induced mucus production in diabetic sensitized animals.

Herein, we also demonstrated that diabetic allergic mice showed a reduction in the levels of IL-5, CCL-11/eotaxin-1, and CCL-3/MIP-1*α* in the lung tissue when compared to those of nondiabetic sensitized challenged mice. This is in line with the assumption that IL-5 and CCL-11/eotaxin-1 together with others T_H_2 cytokines play critical roles in orchestrating and amplifying allergic inflammation in asthma [39]. Furthermore, the decrease in lung eosinophilia of diabetic asthmatic animals can be explained by the lower levels of IL-5, CCL-11/eotaxin, and CCL-3/MIP-1*α* in its lungs, once IL-5 induces terminal maturation of eosinophils and prolongs eosinophil survival by delaying apoptosis death [40]; CCL-11/eotaxin-1 induces mobilization of eosinophils and their progenitors [41]; CCL-3/MIP-1*α* has chemotactic activity for eosinophils, although unlike CCL-11/eotaxin-1, it has no specific chemoattractant activity for eosinophils [42]. The decrease in the levels of IL-5 and CCL-11/eotaxin-1 in the lungs of diabetic sensitized challenged animals could be possibly explained by a drastic reduction of serum insulin levels which may reflect a modulation of T cells in two ways. Insulin was shown to be able to directly reduce CD4^+^ T cell apoptosis and to induce T cell differentiation promoting a shift toward a T_H_2 profile response [43]. Also, alloxan-induced diabetes causes a reduction in the mast cell population, reversed by insulin treatment [24]. In line with our previous observations, the increased levels of corticosterone in diabetic mice could contribute to the reduction in the cytokine production in diabetic allergen-challenged mice [44].

High levels of specific IgE-mediated sensitization to the antigen are frequently observed in asthmatic patients [45]. In these cases, IgE antibodies bind to and crosslink Fc*ε*RI receptors present in mast cell surfaces, leading to degranulation and release of mediators that elicit asthmatic reactions in susceptible individuals [46]. Our data showed that augmentation in OVA-specific serum IgE levels was suppressed in diabetic sensitized mice. These findings are in line with previous data showing the formation of IgE appeared drastically reduced in alloxan-diabetic mice [47, 48]. IL-4 has been described as a major cytokine inducer of switching B cells to produce IgE [49]. In addition, peripheral and thymic T cells from spontaneous nonobese diabetic (NOD) mice [50] secreted significantly less IL-4 compared to cells from their respective controls. In addition, this reduction in OVA-specific serum IgE levels noted in diabetic sensitized mice presents a strong probability of being associated with the high circulating levels of corticosterone, since we previously showed that bilateral adrenalectomy significantly inhibited the drop in the ovalbumin-specific serum IgE concentrations noted in diabetic rats [47].

We further approached the influence of the diabetic state upon the OVA-induced AHR in actively sensitized mice and noted that diabetic mice showed a lower AHR to methacholine after OVA challenge compared to nondiabetic mice. We believed that reduction of eosinophil accumulation in the airways of diabetic mice, after antigen stimulation, could importantly contribute to the lower AHR, as it has been demonstrated a causal relationship between eosinophilic airway inflammation and AHR [51]. However, this hypothesis is controversial since the participation of eosinophils in the development of AHR is not yet completely clarified. Several studies have described a marked inhibition of airway eosinophilia without a concomitant reduction in AHR in mice [52–54]. One possible explanation for our results is that the contribution of eosinophils to the development of AHR in mice might be masked by coexisting pathways that operate independently of eosinophils. Mayr and coworkers proposed that there is a synergism between Fc*ε*RI activation and eosinophils in the induction of AHR in murine models of asthma, once mast cells and eosinophils secrete different mediators that may activate bronchial smooth muscle through distinct pathways, and these pathways cooperate synergistically in inducing AHR [55]. We demonstrated herein that diabetic mice sensitized and challenged with OVA showed a decrease in the serum IgE levels. Thus, it can be hypothesized that the reduction in the AHR in diabetic mice sensitized and challenged with OVA might be dependent on the decrease in airway eosinophilia and mast cell activation. In addition, in our study, diabetic mice showed a reduction in the mucus formation after challenge with OVA, and the obstruction of airways caused by mucus hypersecretion is another important feature of AHR in asthmatics [56].

Type 1 diabetic patients frequently present a hyperactivity of the HPA axis followed by hypercorticoidism [57, 58]. We and others showed that type 1 diabetes induced an increase in circulating corticosterone levels in parallel with an increase of the adrenal gland/body weight rate and expression of adrenocorticotropic hormone (ACTH) receptor in adrenals [21, 59]. We previously noted that the blockade of glucocorticoid receptors restored allergen-induced mast cell activation and eosinophil accumulation in the pleural cavity of diabetic rats [16], besides reestablishing the ability of diabetic rats in producing IgE [47]. Altogether, these findings reinforce the hypothesis that glucocorticoids may be involved with the refractoriness of diabetic mice to develop allergic asthma.

## 5. Conclusion

Our data show that alloxan diabetes leads to a decrease in serum-specific IgE levels and, therefore, to a lower mast cell activation, which may account for the reduced airway inflammation, especially eosinophil recruitment, noted in sensitized and allergen-challenged mice. In addition, the less intense allergen-induced inflammatory response in alloxan-diabetic mice may be responsible for the reduction in AHR.

## Figures and Tables

**Table 1:** Alloxan-induced diabetes causes hyperglycemia, loss of body weight, hypoinsulinemia, and hypercorticoidism.

Group	Glycemia (mmol/L)	Body weight (g)	Plasma insulin (*μ*U/mL)	Plasma corticosterone (ng/mL)
Nondiabetic	6 ± 0.5	36 ± 1	36 ± 7	56 ± 16
Diabetic	23 ± 0.8^+++^	27 ± 1^+++^	8 ± 2^++^	358 ± 44^+++^

Diabetes was induced by a single intravenous injection of alloxan (65 mg/kg) and the analyses performed 27 days after diabetes induction. Values represent mean ± SEM of 6 animals. ^++^*P* < 0.01 compared to nondiabetic mice. ^+++^*P* < 0.001 compared to nondiabetic mice.
